# Supplementary material for: Prevalence, age of decision, and interpersonal warmth judgements of childfree adults: Replication and extensions
Source: PLoS One. 2023 Apr 5;18(4):e0283301. doi: 10.1371/journal.pone.0283301 (PMC10075426; doi:10.1371/journal.pone.0283301)
Supplement: S1 File — (PDF) [file pone.0283301.s001.pdf]

Supporting Information for:
"Prevalence, age of decision, and interpersonal warmth judgements of childfree adults: Replication and extensions"

Jennifer Watling Neal and Zachary P. Neal

S1 Prevalence of reproductive statuses by subgroups

In the manuscript, Figure 2 illustrates the prevalence of each reproductive status within subgroups defined by sex, race, age, education, income, relationship status, and LGBTQIA identity. The text reports the prevalence of childfree adults within each category of each subgroup, and tests for differences. For the sake of completeness, Table S1 presents the estimated prevalence of each reproductive status within each category of subgroups defined by sex, race, age, education, income, relationship status, and LGBTQIA identity.

Subgroup*	Childfree	Parent	NYP	Undecided	Childless	Ambivalent
Sex (χ^2 = 4.53 p = 0.03)						
Men	23.82 (2.4)	47.2 (2.9)	12.55 (2.36)	8.04 (1.67)	4.98 (1.21)	3.4 (0.97)
Women	18.2 (1.79)	58.11 (2.45)	10.49 (1.65)	6.86 (1.18)	3.6 (1.03)	2.75 (1.03)
Race (χ^2 = 6.49 p = 0.01)						
White	22.75 (1.7)	53.62 (2.08)	9.48 (1.48)	7.49 (1.16)	4.03 (0.77)	2.62 (0.75)
Non-White	14.32 (2.91)	49.76 (4.64)	18.85 (3.82)	7.23 (2.08)	5.14 (2.37)	4.7 (1.82)
Age (χ^2 = 0.31 p = 0.58)						
Under 40	19.65 (2.96)	24.09 (3.15)	35.69 (3.88)	17.24 (2.73)	1.86 (0.88)	1.47 (0.93)
40+	21.48 (1.71)	64.75 (2.08)	1.41 (0.54)	3.35 (0.84)	5.28 (1.05)	3.74 (0.92)
Education (χ^2 = 1.04 p = 0.31)						
College grad	23.13 (2.45)	51.49 (3.03)	10.39 (1.96)	7.55 (1.46)	4.08 (1.2)	3.36 (1.09)
Not grad	20.13 (1.82)	53.27 (2.37)	11.9 (1.82)	7.39 (1.28)	4.34 (0.99)	2.96 (0.88)
Income (χ^2 = 2.82 p = 0.09)						
Above median	18.17 (2.1)	57.93 (2.86)	11.12 (2.24)	7.63 (1.53)	3.06 (0.87)	2.1 (0.74)
Below median	22.77 (2.04)	49.41 (2.54)	11.74 (1.86)	7.31 (1.35)	5.07 (1.18)	3.71 (1.07)
Relationship status (χ^2 = 22.02 p < 0.01)						
Ever partnered	17 (1.54)	68.16 (2)	5.03 (0.94)	3.31 (0.73)	4.09 (0.94)	2.41 (0.8)
Always single	31.03 (3.48)	13.54 (2.35)	27.97 (3.94)	17.96 (2.95)	4.75 (1.45)	4.74 (1.48)
LGBTQIA Identity (χ^2 = 24.07 p < 0.01)						
Non-LGBTQIA	18.69 (1.54)	56.03 (2.03)	10.78 (1.51)	6.91 (1.04)	4.48 (0.86)	3.12 (0.77)
LGBTQIA	39.48 (5.76)	26.62 (6.4)	19.19 (5)	10.62 (3.97)	2.78 (1.82)	1.31 (0.93)

* Test of difference in childfree prevalence between two subgroups

Table S1: Prevalence of reproductive statuses within demographic subgroups

S2 Prevalence reproductive statuses in four-category classifications of age and education

The manuscript compares the prevalence of childfree adults by age (under 40 vs. 40+) and by education (College graduate vs. non-graduate). Here, we expand these analyses by considering four-category classifications of age and education. Figure S1 and Table S2 report the prevalence of each reproductive status within each age-based subpopulation. Figure S2 and Table S2 report the prevalence of each reproductive status with each education-based subpopulation.

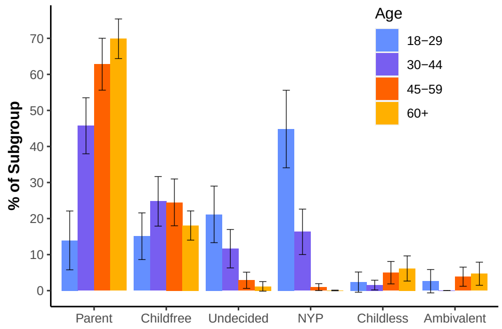

Figure S1: Prevalence of reproductive statuses within age-based subgroups

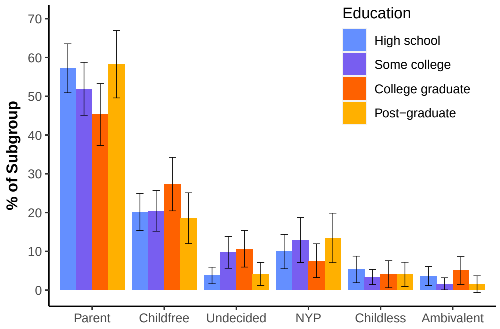

Figure S2: Prevalence of reproductive statuses within education-based subgroups

Subgroup	Childfree	Parent	NYP	Undecided	Childless	Ambivalent
Age						
18-29	15.09 (3.31)	13.94 (4.16)	44.84 (5.49)	21.15 (4)	2.36 (1.43)	2.61 (1.65)
30-44	24.77 (3.51)	45.74 (3.97)	16.31 (3.22)	11.64 (2.72)	1.54 (0.69)	0 (0)
45-59	24.49 (3.31)	62.82 (3.68)	0.97 (0.49)	2.87 (1.15)	4.98 (1.59)	3.86 (1.35)
60+	18.06 (2.07)	69.88 (2.81)	0.05 (0.05)	1.18 (0.68)	6.15 (1.78)	4.69 (1.63)
Education						
High school	20.13 (2.44)	57.21 (3.22)	9.94 (2.26)	3.78 (1.1)	5.33 (1.75)	3.62 (1.25)
Some college	20.43 (2.67)	51.93 (3.48)	12.92 (2.95)	9.74 (2.09)	3.36 (1)	1.62 (0.8)
College graduate	27.34 (3.52)	45.28 (4.06)	7.58 (2.23)	10.65 (2.4)	4.09 (1.78)	5.05 (1.82)
Post-graduate	18.53 (3.34)	58.25 (4.43)	13.46 (3.26)	4.17 (1.51)	4.06 (1.6)	1.52 (1.11)

Table S2: Prevalence of reproductive statuses within age-based and education-based subgroups
